# High overcommitment and low reward as potential predictors for increased depressive symptoms, suicidal ideation, and suicide risk in German veterinarians

**DOI:** 10.1371/journal.pone.0310819

**Published:** 2024-09-24

**Authors:** Kathrin Angelika Schwerdtfeger, Heide Glaesmer, Mahtab Bahramsoltani

**Affiliations:** 1 School of Veterinary Medicine, Institute of Veterinary Anatomy, Freie Universität Berlin, Berlin, Germany; 2 Department of Medical Psychology and Medical Sociology, Medical Faculty, University Medical Center Leipzig, Leipzig, Germany; Centre for Advanced Research Excellence in Public Health, BANGLADESH

## Abstract

Higher rates of depression, suicidal ideation and suicide risk have been reported for veterinarians in Germany. In this study, several demographic and job-related factors were examined to determine whether they could be considered possible predictors of depression, suicidal ideation, and suicide risk. For this purpose, a survey was conducted among veterinarians in Germany. The demographic factors surveyed were gender, age, working status (employed/self-employed), income, field of work (practicing/non-practicing veterinarian), weekly working hours and community size. For assessing job-related factors, the Effort-Reward-Imbalance questionnaire (effort, reward, overcommitment), several subscales of the Copenhagen Psychosocial Questionnaire (quantitative demands, emotional demands, demands for hiding emotions, meaning of work, work-privacy-conflict, thoughts of leaving the job) and the Copenhagen Burnout Inventory were used. A hierarchical logistic regression analysis was performed with the demographic and job-related factors as independent variables and depressive symptoms, suicidal ideation, and suicide risk as dependent variables, respectively. A total of 3.118 veterinarians (78.8% female) between 22 and 69 years (mean age 41.3 years) were included in the study. The factors used resulted in the highest variance explanation for depressive symptoms (57%), followed by suicidal ideation (34%) and suicide risk (23%). Low reward and high overcommitment were found to be the most important predictors of depressive symptoms, suicidal ideation, and suicide risk. Significant relationships with depressive symptoms, suicidal ideation, and suicide risk were also found for burnout, demands for hiding emotions, and thoughts of leaving the job. The results of this study point to opportunities for changes in the veterinary working environment, for the development of prevention and intervention programs for veterinarians, and for the further development of the veterinary curriculum to strengthen the mental health of veterinarians in Germany.

## Introduction

Over the last two decades it has become evident that veterinarians have an up to 4fold risk of suicide compared to the general population [1–3]. Studies that investigated suicide rates between several occupational groups, as well as among healthcare professionals, show a significantly higher suicide rate among veterinarians, even compared to other healthcare professionals [4–6]. While such data are lacking for Germany, a recent large-scale study provided evidence for substantially increased rates of depression, suicidal ideation, and suicide risk in veterinarians in Germany compared to the general population [7]. Veterinarians in Germany were about three times more likely to be screened positive for depression and two times more likely to report about suicidal ideation. Moreover, using the Suicide Behaviors Questionnaire (SBQ-R) [8, 9], about 32% of veterinarians were classified with increased suicide risk, compared to not even 7% in the general population. After controlling for age and gender this is a six to seven times higher suicide risk in veterinarians compared to the general population [7]. While the increased suicide risk in veterinarians is well documented across different studies and geographical regions, less is known about the specific risk factors contributing to this phenomenon. Beside the general risk-factors of suicidal ideation and behavior [10], specific risk factors in veterinarians have been addressed in recent debates and research such as job stressors [2, 11], personality traits [2], access to and knowledge about lethal medications [12–14] and unique work experiences such as euthanasia [15–17]. Among those work-related stressors long working hours, social isolation, feelings of incompetence and treatment errors are the most frequently discussed risk factors that may trigger mental distress and may lead to depression as well as suicidal ideation and behavior [3]. A structured review investigated studies over the last ten years with findings about relationships between job stressors and suicidal ideation and behaviors [18]. They found that exposures to different job stressors are associated with elevated risk of suicide ideation and behavior. Job stressors in combination with emotional work (e.g. the need to show or hide certain emotions) were found as predictors for burnout [19]. The Effort-reward-imbalance (ERI) model addresses the development of stress in the work context where the basic assumption is that employees expect quid pro quos such as salary, appreciation, security, and opportunities for advancement in exchange for their work [20]. It assumes that gratification crises occur when the balance between work effort and reward is not even. In the long term, this could lead to mental and physical illnesses [20]. Connections were found between depression [21, 22] burnout [23] and suicidal ideation [24]. A study investigated the working conditions and work-related satisfaction among German veterinarians in 2017, finding long working hours, low income particularly in recent graduates and low satisfaction regarding key elements of a work-life balance such as leisure time, family time or time spent with administrative tasks. The low monetary compensation paired with high demands regarding long working hours, customer satisfaction and pressure to be omniscient could lead to higher stress levels [25]. A survey of veterinarians in Germany in 2020 showed an improvement in working conditions only in the field of small animal medicine. However, the salaries of veterinarians were still significantly lower than in other academic professions [26]. An online survey of veterinarians in Germany conducted from 2021 to 2023 confirmed a low level of well-being among veterinarians, indicating that the working conditions of veterinarians have not changed substantially to date [27]. These findings have led to several studies being conducted to identify job-related factors that primarily contribute to the low level of well-being among veterinarians [28–30]. For veterinarians in Germany, however, there is only one study to date on job-related factors among official veterinarians [31].

This study aimed to investigate the association of work-related stressors with depressive symptoms, suicidal ideation, and suicide risk in German veterinarians to contribute to our understanding of the specific risk factors of suicidality in veterinarians and to provide clues future for preventive measures. The following hypotheses were purposed.

Low income and high working hours are associated with depressive symptoms, suicidal ideation, and suicide risk in veterinarians.

High effort, low reward and high overcommitment are associated with depression, suicidal ideation, and suicide risk in veterinarians.

High quantitative demands, high emotional demands and demands for hiding emotions are associated with depressive symptoms, suicidal ideation, and suicide risk in veterinarians.

Low meaning of work, thoughts of leaving the job and high work-privacy-conflict are associated with depressive symptoms, suicidal ideation, and suicide risk in veterinarians.

Burnout is associated with depressive symptoms, suicidal ideation, and suicide risk in veterinarians.

## Participants, materials and methods

### Participants

Participants were recruited between July 1 and November 1, 2016 [7]. The call for participation including the link to the web-based questionnaire was published in an article in the July issue of the magazine of the German chamber of veterinarians, which is sent monthly to all veterinarians in Germany. The article included information on the purposes of the study, and that the data would be collected anonymously. In addition, the call for participation was distributed via social media in two German veterinarian Facebook groups and via emails to the 17 federal chambers of veterinarians with the request for distribution among their members via newsletter or other information channels. Furthermore, the call for participation was sent out to the associations of practitioners, employed veterinarians and official veterinarians. A reminder was sent out seven days before the end of the survey via the same channels. Flyers were also included in the conference material of two large national conferences. After the participants had been fully informed about the objectives of the study and the anonymous nature of the data collection, informed consent was obtained from the participants before they started to answer the questionnaire. The questionnaire was conducted via the website program ‘SoSci Survey’. Inclusion criteria were sufficient knowledge of the German language and a completed degree in veterinary medicine. Participants needed about 15–20 minutes to answer all questions over nine screens. Except for the demographic variables on age, gender and income all questions were mandatory. However, participants had the option of selecting “not applicable” if they did not consider a question to be relevant. To preserve anonymity, IP or e-mail addresses were not collected. Thus, multiple participation cannot be ruled out. However, as the participants did not receive any incentives for taking part in the survey, multiple participation can be considered rather unlikely. A total of 3,179 German veterinarians (78.8% female, mean age 41.3 years) answered the questionnaire which represents 7.84% of all veterinarians in Germany at that time. Those with incomplete or unreliable information about age and gender (n = 20), those below the age of 22 (n = 5) and above the age of 69 years (n = 36) were excluded from the analysis. Thus, the data of a sample of 3,118 veterinarians was analysed in the studies [7]. The study protocol was approved by the ethical review board of the Medical Faculty of the University of Leipzig (Az. 110-16-14032016).

### Demographic data

Several demographic data were collected. A detailed description of the local distribution in Germany, the age and the occupational status of the veterinarians as well as a comparison with the entire population of veterinarians in Germany has been published in 2020 [7]. In the questionnaire participants were asked to enter their birth year. The age was later grouped in intervals of 10 years. The occupational status had the following options: being self-employed or employed, being unemployed (by choice or currently looking for a job), parental leave, working in a non-veterinarian field, retired, and incapacitated for employment. For the analysis only two variables, being self-employed or employed, were used. The field of work included 14 options (practicing veterinarian in the fields of small animal medicine, horses, farm animals, mixed practice, food hygiene in general or in slaughterhouse, industry with the options of research & development, technical service or field service, public health, research, university, zoo animals and other) with the possibilities to choose more than one. They were coded in two groups, practicing veterinarians and non-practicing veterinarians, since there are distinct differences between practicing and non-practicing veterinarians, especially regarding the working conditions (e.g. working hours, night and weekend working, vacation days, salary) [26] and the role of practicing veterinarians as caregivers [32, 33]. The level of income was specified to be net income through the veterinarian profession. Possibilities to answer were in steps ranging from below €1,000, going in €1,000 steps up to below €5,000 and finally over €5,000. There was also the option of not providing any information on income. Respondents were asked for their community size, classified in below 5,000, below 20,000, below 100,000 and over 100,000 inhabitants. Working hours were assessed with an open question, asking for all hours including overtime hours per week. They were later grouped in 0–20, 21–40, 41–60, 61–80 and more than 80 working hours per week. If the participants stated a range (such as 50–60 hours), the arithmetic mean was calculated.

### Instruments

#### PHQ -9

The Depression module of the Patient Health Questionnaire (PHQ-9) was used to assess symptoms of depression, since it shows very good psychometric properties, including sensitivity and specificity to clinical interviews (gold standard) [34, 35]. The PHQ-9 consists of nine items assessing depressive symptoms according to DSM-IV. Response categories for these items range from 0 (“not at all”) to 3 (“nearly every day”), thus the total scores range between 0 and 27. The total score of the responses suggests varying levels of depressive symptoms: 0–4 = none to minimal, 5–9 = mild, 10–14 = moderate, 15–19 = moderately severe and 20–27 = severe depressive symptoms. A cut-off of 10 showed sensitivity and specificity of 88% each, for major depressive disorders in the original validation study [36] and is therefore widely used and accepted [37]. Thus, a total score of 10 and above was used to identify participants with relevant depressive symptoms. Moreover, item 9 (“Thoughts that you would be better off dead, or of hurting yourself in some way”) was used to assess suicidal ideation. Participants were classified as having current suicidal ideation if item 9 was answered with scores from 1 (“several days”) to 3 (“nearly every day”) [34, 35].

#### SBQ-R

The Suicide Behaviors Questionnaire-Revised (SBQ-R) [8, 9] was developed to economically assess different aspects of suicidality. It consists of four items assessing suicidal ideation or attempt, frequency of suicidal ideation over the past twelve months, threat of suicidal attempt, likelihood of suicidal behavior in the future. The total score of the four items ranges from 3 to 18. A cut-off score of 8+ was established for identification of people with an increased risk of suicide [8]. This cut-off score was also determined with the help of ROC analyses [38]. Therefore, a score of 8 and above was used to identify patients with increased suicide risk. Internal consistencies of the SBQ-R are α ≥.76 across several studies [8, 9].

#### Effort-Reward-Imbalance Questionnaire

The Effort-Reward-Imbalance Questionnaire (ERI) [20–22] is a well-established instrument assessing reciprocity between efforts made in the workplace and rewards received for one’s efforts. It measures three different aspects, namely ‘effort’, ‘reward’ and ‘overcommitment’. ‘Effort’ is measured by three items, referring to the demanding aspects of the work environment including time pressure, frequent work interruptions and increasing workload. Seven items are used to measure ‘reward’ perceived by the person, referring to salary, appreciation, career opportunities and job security. Six items are used to measure aspects of work-related ‘overcommitment’, referring to excessive motivation of the person to meet the work requirements. Numerous studies have shown that high effort in combination with low reward as well as a high level of overcommitment lead to increased stress levels with negative consequences for mental and physical health [39]. All questions refer to the current workplace and participants are asked to indicate in how far the items reflect their typical work situation. The items are rated on a 4-point Likert scale ranging from (1) strongly disagree, (2) disagree, (3) agree to (4) strongly agree. Higher scores represent higher ‘effort’, ‘reward’ or rather ‘overcommitment’. Cronbach’s α coefficients are equal to or higher than 0.80 (effort = 0.80, reward = 0.84, overcommitment = 0.85) [9].

#### Copenhagen Psychosocial Questionnaire

The Copenhagen Psychosocial Questionnaire (COPSOQ) was originally developed as a screening instrument to assess work-related mental stress and strain [40]. It has been revised in 2005 (COPSOQ II) and again in 2019 (COPSOQ III) [41]. The German version of COPSOQ II was validated in 2006 [42]. From the 41 different subscales of the German version of COPSOQ II, the subscales ‘quantitative demands’ (4 items), ‘emotional demands’ (3 items), ‘demands for hiding emotions’ (2 items), ‘meaning of work’ (3 items) as well as the implemented pre-existing instruments work-family-conflict scale (5 items) [43] and self-rated health (one item) from the NEXT study (‘thoughts of leaving the job’) have been used in this study. These subscales were selected because, on the one hand, a high workload, particular emotional demands and negative work-home interactions were identified as relevant stressors for veterinarians [44] and, on the other hand, the veterinary profession appears to be perceived as predominantly meaningful by veterinarians [45]. In addition, the Copenhagen Burnout Inventory (CBI) (6 items) was used [40]. The questions of the COPSOQ II used in this study have 5-point Likert scales (ranging from “Always”, “Often”, “Sometimes”, “Seldom” to “Never” or from”Hardly ever/To a very large extent”,”To a large extent”, “Somewhat”, “To a small extent”, to “To a very small extent”). All the scales of COPSOQ are scored 0–100 points. The five response options are scored 100, 75, 50, 25, 0. For all scales, high scores correspond to high values on the respective dimension. The work-family-conflict scale (WFC) is one of two scales developed by Netemeyer et al. 1996 for better generalization of work-family-conflict [43]. The original WFC scale consists of five questions but was later reduced to four due to validity reasons [46]. The WFC scale is used in the German COPSOQ as a five items scale with five possible answers (“Yes, often”, “Yes, sometimes”, “Not decided”, “Rarely”, “No, never”*)* and the term ‘family’ was extended to ‘private life’ [47]. The CBI is an instrument for assessing symptoms of the burnout syndrome, including the subscales personal burnout, work-related burnout, and client-related burnout [40]. It has been validated with the PUMA study (Project on Burnout, Motivation and Job Satisfaction) and its reliability has been proven. As the German COPSOQ only includes the subscale personal burnout, this subscale was applied within this study. It consists of five items with a 5-point Likert scale (“Yes, often”, “Yes, sometimes”, “Not decided”, “Rarely”, “No, never”) to measure personal exhaustion and vulnerability. As with the COPSOQ subscales, the five response options are scored as 100, 75, 50, 25 and 0 [47].

### Statistical analysis

Hierarchical logistic regression analyses were used to determine the extent to which the demographic factors, the reciprocity between effort and rewards, work-related psychosocial stressors and burnout predict depressive symptoms (PHQ-9 score ≥ 10), suicidal ideation (PHQ-9, Item 9 score > 0), or suicide risk (SBQ-R score ≥ 8). In model 1 the predictive value of the demographic factors was tested. In model 2 the additional predictive value of effort, reward and overcommitment (ERI subscales) was tested. Model 3 tested the additional predictive value of work-related psychosocial stressors (COPSOQ subscales), and model 4 tested the additional predictive value of Burnout assessed with the CBI. The models were designed to test the hypotheses according to theoretical assumptions that the subscales of the respective questionnaires are associated with each other with the ERI focusing on the balance between effort and reward [39], while the COPSOQ subscales address the spectrum of psychosocial work strains [42] and the CBI, as a modular subscale, measures the outcome of these strains [48]. The analyses were conducted with IBM SPSS Statistics. The significance level was set at 5%. Multicollinearity was addressed in two ways. The intercorrelations of all included variables were tested. These were all below 0.8 and it can therefore be assumed that there is no multicollinearity problem. In addition, the standard errors of the regression coefficients were inspected. These are all relatively low and in a similar range. This also speaks against multicollinearity.

## Results

### Demographics and descriptive statistics

The data on gender, age and work status included in the analysis in this study were already part of a previous publication [7]. In the sample of the 3,118 veterinarians (79.5% female), 486 participants were in the 22–29 year-old group (88.3% female), 1,119 in the 30–39 year-old group (90.0% female), 754 in the 40–49 year-old group (81.2% female), 591 in the 50–59 year-old group (61.8% female) and 168 in the 60–69 year-old group (38.7% female) [7]. Among the 2,894 veterinarians who stated that they worked in a veterinary profession, 883 participants were self-employed (66.5% female) and 2,011 participants were employed (88.7% female) [7].

Other demographic factors included in the analysis were income, field of work, weekly working hours and community size (Table 1). Most of the veterinarians surveyed had a net income of between €2.000 and €5.000 per month (female: 48.2%; male: 57.6%) and the majority worked as practicing veterinarians (female: 63.6%; male: 58.0%). Most of them worked between 40 and 59 hours per week (female: 53.9%; male: 58.5%). The majority of participants lived either in rural areas with fewer than 5,000 inhabitants (female: 30.9%; male: 35.2%) or in large cities with more than 100,000 inhabitants (female: 32.8%; male: 27.2%) (Table 1).

**Table 1:** Sociodemographic characteristics of the sample.

	Female		Male		Total	
	N	%	N	%	N	%
**Income**						
<€1,000	243	10.3	25	4.1	268	9.1
€1,000-€2,000	893	38.2	74	12.2	967	32.8
€2,000-€5,000	1,126	48.2	349	57.6	1,475	50.1
>€5,000	76	3.3	158	26.1	234	8,0
Total	2,338	100	606	100	2,944	100
**Field of work**						
practicing veterinarians	1,576	63.6	371	58.0	1,947	62.4
non-practicing veterinarians	902	36.4	269	42.0	1,171	37.6
Total	2,478	100	640	100	3,118	100
**Weekly working hours**						
0–19	80	3.4	6	1.2	86	2.9
20–39	545	23.0	52	8.2	597	19.9
40–59	1,279	53.9	369	58.5	1,648	54.9
60–79	359	15.1	173	27.4	532	17.7
≥80	108	4.6	31	4.9	139	4.6
Total	2,371	100	631	100	3,002	100
**Community Size**						
<5,000 inhabitants	765	30.9	235	35.2	1,000	31.8
<20,000 inhabitants	500	20.2	152	22.8	652	20.7
<100,000 inhabitants	398	16.1	99	14.8	497	15.8
>100,000 inhabitants	812	32.8	182	27.2	994	31.7
Total	2,475	100	668	100	3,143	100

The results of the ERI questionnaire, which can range from 3.00 to 12.00, show that females (8.63) and males (8.64) reported a similar level of ‘effort’. For ‘reward’ (range 7.00–28.00) the value was higher for males (18.53) than for females (17.38). ‘Overcommitment’ (range 06.00–24.00) was more prevalent among female veterinarians (16.17) than among male veterinarians (15.00). For the COPSOQ subscales, higher values were found for females for the subscales ‘quantitative demands’ (female: 6.19, male: 59.65), ‘emotional demands’ (female: 62.78, male: 55.40), ‘demands for hiding emotions’ (female: 50.39, male: 47.11), ‘work-privacy-conflict’ (female: 61.67, male: 56.36), ‘thoughts of leaving the job’ (female: 31.33, male: 25.47) and the CBI (female: 51.86, male: 42.68), while male veterinarians reported a higher value for ‘meaning of work’ (male: 72.20, female: 71.11) (Table 2).

**Table 2:** Descriptive statistics: Effort-Reward-Imbalance Questionnaire (ERI) and Copenhagen Psychosocial Questionnaire (COPSOQ) subscales.

	Min—Max	Female		Male		Total	
		*n* = 640		*n* = 2,477		*n* = 3,118	
		M	SD	M	SD	M	SD
**ERI Subscales**							
Effort	3.00–12.00	8.63	1.80	8.64	1.84	8.63	1.81
Reward	7.00–28.00	17.38	3.89	18.53	3.94	17.62	3.93
Overcomittment	6.00–24.00	16.17	3.35	15.00	3.44	15.93	3.40
**COPSOQ Subscales**							
Quantitative demands	0.00–100.00	61.19	19.00	59.65	19.10	60.88	19.03
Emotional demands	0.00–100.00	62.78	20.07	55.40	20.73	61.27	20.42
Demands for hiding emotions	0.00–100.00	50.39	22.23	47.11	23.80	49.72	22.59
Meaning of work	0.00–100.00	71.11	19.19	72.20	18.91	71.33	19.14
Work-privacy-conflict	0.00–100.00	61.67	26.98	56.36	28.71	60.58	27.43
Thoughts of leaving the job	0.00–100.00	31.33	29.05	25.47	26.75	30.12	28.69
Copenhagen Burnout Inventory (CBI)	0.00–100.00	51.86	19.32	42.68	20.28	49.98	19.87

### Potential predictors for depressive symptoms

If only the demographic variables were included in model 1 of the logistic regression, all but community size were significant. The probability of depressive symptoms increased with higher age, more working hours, and was higher in male as well as employed veterinarians. Higher income and higher field of work status decreased the probability. In model 1, 7% of the variance were clarified.

After including the three ERI subscales in model 2 the demographic variables were no longer significant. The ERI subscales ‘reward’ and ‘overcommitment’ showed a significant association with depressive symptoms: higher ‘reward’ lowered the probability of depressive symptoms whereas higher ‘overcommitment’ increased this probability. After including the ERI subscales in model 2, 30% of the variance were explained.

Adding the COPSOQ subscales ‘quantitative demands’, ‘emotional demands’, ‘demands for hiding emotions’, ‘meaning of work’, ‘work-privacy-conflict’ and the COPSOQ item ‘thoughts of leaving the job’ in model 3, nothing fundamentally changed for demographic and ERI subscales compared to model 2. ‘Demands for hiding emotions’, ‘work-privacy-conflict’, and ‘thoughts of leaving the job’ were significantly and positively associated with depressive symptoms. In turn, ‘meaning of work’ was significantly negatively associated with depressive symptoms. However, after including these COPSOQ subscales in model 3 further 8% of the variance were explained.

After adding the CBI in model 4, the results for demographic and ERI subscales remained unchanged. The COPSOQ subscales ‘demands for hiding emotions’, ‘meaning of work’ and ‘thoughts of leaving the job’ remained as significant predictors as in model 3, but ‘work-privacy-conflict’ was no longer a significant predictor, ‘emotional demands’ were now a significant predictor with being negatively associated with depressive symptoms. The CBI was significantly associated with depressive symptoms with higher CBI scores being positively associated with depressive symptoms. The inclusion of the CBI contributed further 12% of variance explanation.

Together, 57% of the variance in depressive symptoms could be explained by the factors included in this analysis (Table 3).

**Table 3:** Hierarchical logistic regression analysis: Depressive symptoms.

Variable	Model 1			Model 2			Model 3			Model 4		
	B	OR (CI)	p	B	OR(CI)	p	B	OR(CI)	p	B	OR(CI)	p
Constant	-2.36	0.09	< .001***	-3.22	0.04	< .001***	-4.36	0.01	< .001***	-6.62	0.00	< .001***
Age	0.02	1.02 (1.00–1.03)	.009**	0.00	1.00 (0.99–1.02)	.609	0.01	1.01 (1.00–1.03)	.200	0.01	1.01 (0.99–1.03)	.202
Income	-0.42	0.66 (0.57–0.77)	< .001***	-0.07	0.94 (0.78–1.13)	.490	-0.15	0.86 (0.71–1.04)	.128	-0.14	0.87 (0.70–1.07)	.192
Gender	-0.54	0.58 (0.44–0.76)	< .001***	-0.25	0.78 (0.57–1.07)	.120	-0.25	0.78 (0.55–1.09)	.142	0.04	1.04 (0.71–1.51)	.847
Field of work	-0.43	0.65 (0.51–0.83)	.001**	-0.15	0.86 (0.65–1.14)	.287	-0.01	0.99 (0.71–1.37)	.932	-0.12	0.89 (0.62–1.27)	.520
Work status	0.37	1.45 (1.11–1.91)	.008**	0.01	1.01 (0.73–1.38)	.974	-0.13	0.88 (0.63–1.23)	.446	0.01	1.01 (0.69–1.46)	.981
Working hours	0.56	1.75 (1.53–2.01)	< .001***	0.09	1.10 (0.93–1.29)	.271	0.00	1.00 (0.83–1.20)	.990	0.05	1.05 (0.86–1.28)	.628
Community size	0.05	1.05 (0.97–1.13)	.250	0.06	1.07 (0.97–1.17)	.164	0.09	1.09 (0.99–1.20)	.077	0.08	1.09 (0.98–1.21)	.122
ERI: Effort				0.01	1.01 (0.94–1.09)	.834	-0.04	0.96 (0.88–1.06)	.443	-0.08	0.92 (0.83–1.02)	.113
ERI: Reward				-0.19	0.82 (0.80–0.85)	< .001***	-0.08	0.92 (0.89–0.96)	< .001***	-0.05	0.95 (0.91–0.99)	.015*
ERI: Overcommitment				0.32	1.37 (1.31–1.43)	< .001***	0.25	1.29 (1.23–1.36)	< .001***	0.17	1.19 (1.13–1.26)	< .001***
COPSOQ: Quantitative demands							0.00	1.00 (0.99–1.01)	.797	-0.01	0.99 (0.98–1.00)	.184
COPSOQ: Emotional demands							0.00	1.00 (0.99–1.01)	.855	-0.01	0.99 (0.98–1.00)	.005**
COPSOQ: Demands for hiding emotions							0.01	1.01 (1.01–1.02)	< .001***	0.01	1.01 (1.00–1.02)	.001**
COPSOQ: Meaning of work							-0.02	0.98 (0.98–0.99)	< .001***	-0.01	0.99 (0.98–1.00)	.001**
COPSOQ: Work-Privacy-Conflict							0.01	1.01 (1.01–1.02)	< .001***	0.01	1.01 (1.00–1.01)	.225
COPSOQ: Thoughts of leaving the job							0.02	1.02 (1.01–1.02)	< .001***	0.01	1.01 (1.01–1.02)	< .001***
COPSOQ: CBI										0.09	1.09 (1.08–1.10)	< .001***
R^2^/ ΔR^2^	.07/.07			.37/.30			.45/.08			.57/.12		

*Notes*. Gender: 1 = male, 2 = female; Work status: 1 = self-employed, 2 = employed; Field of work: 1 = practicing veterinarians, 2 = non-practicing veterinarians

*p < .05,

**p < .01,

***p < .001

### Potential predictors for suicidal Ideation

Including all demographic variables in model 1, there was a significant association of age, income, and working hours with suicidal ideation. The probability of suicidal ideation was higher with higher age and more working hours, while lower with higher income. Gender, field of work, work status and community size had no significant effect. The demographic variables explained 4% of the variance.

Once the three ERI subscales were added in model 2, none of the demographic variables remained significant, while the three ERI subscales were significant. Higher values for ‘effort’ and ‘reward’ were related to lower probabilities of suicidal ideation, and higher ‘overcommitment’ was associated with a higher probability of suicidal ideation. The inclusion of the ERI subscales led to additional 17% of the variance explained.

By adding the COPSOQ items in model 3, no significant changes in the demographic and ERI variables were found. The COPSOQ subscales ‘demands for hiding emotions’ and ‘thoughts of leaving the job’ were significantly associated with the probability of suicidal ideation, both were positively associated with suicidal ideation; thus, higher ‘demands for hiding emotions’ and ‘thoughts of leaving the job’ increased the likelihood on suicidal ideation. The COPSOQ subscales ‘quantitative demands’, ‘emotional demands’, ‘meaning of work’, and ‘work-privacy-conflict’ had no significant association with suicidal ideation. With the COPSOQ subscales, an additional 5% of the variance were explained.

By adding the CBI in model 4, another 8% of the variance were explained. In this model, gender was a significant predictor, with female veterinarians to be more likely than male veterinarians to report about suicidal ideation. The three ERI subscales remained significant and there was no change in the findings for the COPSOQ subscales. The CBI was significantly and positively associated with suicidal ideation.

Overall, 34% of the variance in suicidal ideation were explained by these factors (Table 4).

**Table 4:** Hierarchical logistic regression analysis: Suicidal ideation.

Variable	Model 1			Model 2			Model 3			Model 4		
	B	OR (CI)	p	B	OR(CI)	p	B	OR(CI)	p	B	OR(CI)	p
Constant	-2,87	0.06	< .001***	-2.35	0.10	.002**	-3.56	0.03	< .001***	-4.76	0.01	< .001***
Age	0.02	1.02 (1.00–1.03)	.016*	0.01	1.01 (0.99–1.02)	.363	0.01	1.01 (0.99–1.02)	.241	0.01	1.01 (1.00–1.03)	.195
Income	-0.28	0.76 (0.64–0.90)	.001**	0.10	1.10 (0.91–1.34)	.317	0.06	1.06 (0.87–1.29)	.556	0.10	1.10 (0.90–1.35)	.352
Gender	-0.03	0.97 (0.73–1.28)	.082	0.24	1.28 (0.94–1.74)	.123	0.29	1.34 (0.97–1.85)	.076	0.53	1.70 (1.21–2.39)	.002**
Field of work	-0.27	0.76 (0.58–1.01)	.054	-0.02	0.98 (0.73–1.32)	.884	0.08	1.09 (0.78–1.52)	.625	0.00	1.00 (0.70–1.42)	.997
Work status	0.12	1.13 (0.84–1.53)	.425	-0.16	0.85 (0.61–1.18)	.333	-0.23	0.79 (0.56–1.12)	.181	-0.13	0.88 (0.62–1.26)	.482
Working hours	0.47	1.59 (1.37–1.85)	< .001***	0.14	1.15 (0.98–1.37)	.097	0.13	1.14 (0.95–1.37)	.163	0.17	1.18 (0.98–1.42)	.085
Community size	0.08	1.08 (0.99–1.18)	.087	0.09	1.09 (0.99–1.20)	.065	0.10	1.11 (1.00–1.22)	.041*	0.10	1.10 (1.00–1.22)	.061
ERI: Effort				-0.12	0.89 (0.82–0.96)	.003**	-0.16	0.85 (0.77–0.94)	.001**	-0.20	0.82 (0.74–0.90)	< .001***
ERI: Reward				-0.16	0.85 (0.82–0.88)	< .001***	-0.08	0.92 (0.89–0.96)	< .001***	-0.06	0.94 (0.90–0.98)	.002**
ERI: Overcommitment				0.22	1.25 (1.20–1.31)	< .001***	0.17	1.18 (1.13–1.25)	< .001***	0.10	1.11 (1.05–1.17)	< .001***
COPSOQ: Quantitative demands							0.00	1.00 (0.99–1.01)	.786	-0.01	1.00 (0.99–1.01)	.328
COPSOQ: Emotional demands							0.00	1.00 (1.00–1.01)	.407	-0.01	1.00 (0.99–1.00)	.279
COPSOQ: Demands for hiding emotions							0.01	1.01 (1.01–1.02)	< .001***	0.01	1.01 (1.00–1.02)	.001**
COPSOQ: Meaning of work							-0.01	0.99 (0.99–1.00)	.122	0.00	1.00 (0.99–1.01)	.782
COPSOQ: Work-Privacy-Conflict							0.00	1.00 (1.00–1.01)	.344	0.00	1.00 (0.99–1.00)	.288
COPSOQ: Thoughts of leaving the job							0.02	1.02 (1.01–1.02)	< .001***	0.01	1.01 (1.01–1.02)	< .001***
COPSOQ: CBI										0.06	1.06 (1.05–1.07)	< .001***
R^2^/ ΔR^2^	.04/.04			.21/.17			.26/.05			.34/.08		

*Notes*. Gender: 1 = male, 2 = female; Work status: 1 = self-employed, 2 = employed; Field of work: 1 = practicing veterinarians, 2 = non-practicing veterinarians

*p < .05,

**p < .01,

***p < .001

### Potential predictors for suicide risk

In model 1, including all demographic variables age, income, and working hours were found to be significant predictors of suicide risk. Higher income decreased the probability of suicide risk, while more working hours and higher age were associated with suicide risk. In this first model, 4% of the variance were explained.

By adding the three ERI subscales in model 2, the income variable became non-significant, while age and working hours remained significant. In contrast to the analysis for suicidal ideation, however, ‘effort’ was not significantly related to suicide risk. Higher values for ‘reward’ were related to lower probability of suicide risk and higher ‘overcommitment’ was related to higher probability of suicide risk. By adding the ERI subscales, additional 9% of variance were explained.

By including the COPSOQ subscales in model 3, ‘reward’ was no longer significant, but ‘overcommitment’ remained significant. The COPSOQ subscales ‘quantitative demands’, ‘demands for hiding emotions’ and ‘thoughts of leaving the job’ were significantly related to suicide risk. Higher ‘quantitative demands’ decreased the likelihood of suicide risk, while higher ‘demands for hiding emotions’ and increased ‘thoughts of leaving the job’ were positively related to suicidal risk. In model 3, another 5% of the variance were explained.

After adding the CBI in model 4, the results for demographic variables remained unchanged. The COPSOQ subscales ‘quantitative demands’, ‘demands for hiding emotions’ and ‘thoughts of leaving the job’ continued to be significant predictors. ‘Overcommitment’ was no longer a significant predictor, while the CBI was significantly and positively related to suicidal risk. Adding the CBI increased the variance explained by another 5%.

Overall, 23% of the variance in suicide risk were explained by these factors (Table 5).

**Table 5:** Hierarchical logistic regression analysis: Suicide risk.

Variable	Model 1			Model 2			Model 3			Model 4		
	B	OR (CI)	p	B	OR(CI)	p	B	OR(CI)	p	B	OR(CI)	p
Constant	-2.03	0.13	< .001***	-1.96	0.14	.001**	-3.31	0.04	< .001***	-4.09	0.02	< .001***
Age	0.02	1.02 (1.01–1.03)	.001**	0.01	1.01 (1.00–1.03)	.0264*	0.02	1.02 (1.00–1.03)	.008**	0.02	1.02 (1.01–1.03)	.004**
Income	-0.30	0.74 (0.64–0.86)	< .001***	-0.09	0.91 (0.78–1.07)	.255	-0.14	0.87 (0.74–1.03)	.096	-0.12	0.89 (0.75–1.05)	.158
Gender	-0.12	0.89 (0.70–1.13)	.324	0.08	1.08 (0.84–1.40)	.536	0.12	1.13 (0.87–1.47)	.356	0.27	1.31 (1.00–1.71)	.052
Field of work	-0.20	0.82 (0.65–1.03)	.086	-0.04	0.96 (0.76–1.22)	.754	0.11	1.12 (0.85–1.46)	.424	0.05	1.05 (0.80–1.39)	.714
Work status	0.07	1.08 (0.83–1.39)	.570	-0.13	0.88 (0.67–1.15)	.332	-0.16	0.85 (0.64–1.12)	.254	-0.11	0.90 (0.68–1.20)	.469
Working hours	0.42	1.52 (1.34–1.73)	< .001***	0.18	1.19 (1.04–1.37)	.014*	0.19	1.21 (1.04–1.41)	.015*	0.23	1.25 (1.07–1.46)	.004**
Community size	0.04	1.04 (0.97–1.12)	.270	0.05	1.05 (0.97–1.13)	.211	0.06	1.06 (0.98–1.15)	.146	0.06	1.06 (0.98–1.15)	.164
ERI: Effort				-0.02	0.98 (0.92–1.05)	.565	-0.02	0.99 (0.91–1.07)	.706	-0.04	0.97 (0.89–1.05)	.389
ERI: Reward				-0.09	0.91 (0.89–0.94)	< .001***	-0.02	0.98 (0.95–1.01)	.245	0.00	1.00 (0.97–1.03)	.860
ERI: Overcommitment				0.14	1.15 (1.11–1.19)	< .001***	0.09	1.09 (1.05–1.13)	< .001***	0.04	1.04 (1.00–1.08)	.087
COPSOQ: Quantitative demands							-0.01	0.99 (0.98–1.00)	.0154*	-0.01	0.99 (0.98–1.00)	.003**
COPSOQ: Emotional demands							0.01	1.01 (1.00–1.01)	.079	0.00	1.00 (0.99–1.01)	.928
COPSOQ: Demands for hiding emotions							0.01	1.01 (1.00–1.01)	.002**	0.01	1.01 (1.00–1.01)	.008**
COPSOQ: Meaning of work							0.00	1.00 (0.99–1.00)	.163	0.00	1.00 (0.99–1.01)	.845
COPSOQ: Work-Privacy-Conflict							0.01	1.01 (1.00–1.01)	.056	0.00	1.00 (1.00–1.01)	.937
COPSOQ: Thoughts of leaving the job							0.02	1.02 (1.01–1.02)	< .001***	0.01	1.01 (1.01–1.02)	< .001***
COPSOQ: CBI										0.04	1.04 (1.03–1.05)	< .001***
R^2^/ ΔR^2^	.04/.04			.13/.09			.18/.05			.23/.05		

*Notes*. Gender: 1 = male, 2 = female; Work status: 1 = self-employed, 2 = employed; Field of work: 1 = practicing veterinarians, 2 = non-practicing veterinarians

*p < .05,

**p < .01,

***p < .001

## Discussion

Numerous studies conducted in various geographical regions indicate different reasons for low mental well-being, higher depression and suicide rates in veterinarians [4–6, 26–31]. Reasons range from personality traits, work-related stressors, easy access to lethal medication, low income, euthanasia distress, alcohol and drug abuse to social and professional isolation [1–3, 7, 26, 28–31, 49–54]. This study investigated the association of several demographic and job-related factors on the probability of depressive symptoms, suicidal ideation, and suicide risk among veterinarians in Germany. The investigations were conducted using hierarchical logistic regression analysis since it provides the opportunity to test the additional explanatory power of the predictor variables sequentially, as each questionnaire addresses a different overarching aspect. While the ERI subscales focus on the balance between effort and reward [39], the COPSOQ subscales measure the spectrum of psychosocial work strains [42] and the CBI the outcome of these strains [48].

With the factors used in the study, the highest variance explained (57%) was found for depressive symptoms. The demographic variables selected for the study are negligible as predictors of depressive symptoms, since they not only contributed very little to the explained variance, but they were also not significant once other variables were added in the models. Even if the several aspects surveyed with the COPSOQ subscales made a rather small contribution to the explained variance in depressive symptoms, the aspects ‘demands for hiding emotions’, ‘meaning of work’ and ‘thoughts of leaving the job’ were consistently important. Veterinarians are often confronted with emotionally challenging situations, such as euthanizing a companion animal, where a professional job performance is not compatible with showing one’s true emotions [55, 56]. Hiding the true emotions results in surface-acting emotional labor, which can lead to depression if it has to be maintained over a long period of time [57]. Other studies also show that the perceived meaningfulness of work is negatively related to depression [58, 59]. Similarly, a meaningful work experience counteracts the desire to leave the job [60, 61]. It is therefore plausible that ‘thoughts of leaving the job’ was positively associated with depressive symptoms. This is also confirmed by several studies carried out in the healthcare sector during the Covid-19 pandemic, which also showed a relationship between the intention to leave the job and depression, whereby depression was assumed to be the cause of the intention to leave the job [62, 63]. Symptoms of burnout assessed with the CBI were positively associated with depressive symptoms, and in contrast to the other COPSOQ subscales, the CBI contributed to a higher explanation of variance for depressive symptoms. This finding is not surprising, because even though burnout and depression are two distinct concepts [64], they share some aspects [65, 66]. Job-related factors that contribute to emotional exhaustion, a major component of burnout assessed with the CBI, include work-home interference and high workload, combined with the emotional stress of interacting with animal owners and being confronted with animal suffering [67, 68]. The ERI subscales ‘reward’ and ‘overcommitment’ explained the highest amount of variance, with ‘reward’ to be negatively and ‘overcommitment’ to be positively related to probability of depressive symptoms. Other studies have also found a higher risk for depression in connection to lower scores in ‘reward’ and higher scores in ‘overcommitment’ [69, 70]. Despite their high effort, veterinarians may perceive lower reward in terms of financial compensation and recognition [25, 71]. Overcommitment is described as a tendency to show excessive devotion to one’s work and the theory postulates, that overcommitment leads to more stress and negative health effects [20, 72, 73]. This has been proven in several studies [74, 75] and is also confirmed by the results of the German veterinarians surveyed. Overcommitment often manifests in veterinarians assuming that they are personally responsible for the well-being of the clients and their animals, far beyond the scope of their duties. With such overcommitment, however, veterinarians are vulnerable to compassion fatigue [76]. Compassion fatigue is a condition that can occur as a result of secondary traumatic stress, which people experience when they witness other people’s traumas as caregivers [77], and is significantly associated with depression [78].

In case of suicidal ideation, the factors included in the study explained 34% of the variance. As with depressive symptoms, the demographic factors were also of no relevance for suicidal ideation, since they only accounted for a very small proportion of the variance explained and none of the factors were consistently significant. The several COPSOQ subscales made an equally small contribution to the explained variance. However, the aspects ‘demands for hiding emotions’ and ‘thoughts of leaving the job’ were positively associated with suicidal ideation. The positive relationship between hiding real emotions through emotional surface-acting and suicidal ideation was also confirmed in a longitudinal study of military personnel [79]. Veterinarians are exposed to numerous stressors in their work, such as long working hours, demanding client expectations and unexpected outcomes, which contribute considerably to the decision to leave the job [80]. High job stress, in turn, is a particular job-related predictor of suicidal ideation [81]. Burnout assessed with the CBI, was a predictor of suicidal ideation, as in the case of depressive symptoms. Burnout contributed slightly more to variance explained in suicidal ideation than the other COPSOQ subscales. Several other studies also point to the relationship between burnout and suicidal ideation [44, 82, 83]. Burnout has a mediating role in the positive relationship between job-related stressors and suicidal ideation [84]. The highest variance explained was found for suicidal ideation with the ERI subscales, with all three subscales, ’effort’, ’reward’, and ’overcommitment’ being associated with suicidal ideation. Interestingly, there was a negative association between ’effort’ and suicidal ideation, a finding that is not confirmed by other studies in which the ERI questionnaire was used to examine the relationship between ‘effort’ and suicidal ideation [24, 85]. The three aspects assessed by the ERI subscale ‘effort’ are time pressure, frequent interruptions at work and an accumulating workload [20]. Studies investigating the relationship of these aspects with suicidal ideation or mental health in veterinarians also found no negative relationship with suicidal ideation or positive relationship with mental health [2, 25, 54, 86]. In contrast, the negative relationship of ‘reward’ as well as the positive relationship of ‘overcommitment’ with suicidal ideation found among the veterinarians surveyed were also confirmed by other studies [2, 24, 85, 87].

The lowest amount of variance explained by the factors used was found for suicide risk (23%). Although the demographic factors also contributed the least to the variance explanation for suicide risk, higher age and longer working hours were positively associated with suicide risk in all models of the logistic regression analysis. Higher age is an established risk factor for suicide risk for decades with numerous studies [88–91], thus the results of this study are in line with previous research. Similarly, studies in specific occupational groups confirm that long working hours contribute to an increased suicide risk [2, 92–94]. Since working hours are often unpredictable in the veterinary profession and very long working hours are indeed often the norm [2, 25, 71], the positive association of long working hours and suicide risk among the veterinarians in this study is understandable. In the COPSOQ subscales, which also contributed only a very small proportion to the variance explained, ’quantitative demands’, ’demands of hiding emotions’, ’thoughts of leaving the job’ and burnout (CBI) were associated with suicide risk. While this relationship was positive for ’demands of hiding emotions’, ’thoughts of leaving the job’ and burnout, as expected, there was a surprisingly negative relationship between ’quantitative demands’ and suicide risk. The questions in the COPSOQ subscale ‘quantitative demands’ mainly relate to workload and time pressure [43]. However, a positive relationship with suicide risk is described for both factors in other studies on physicians and veterinarians [86, 95, 96]. Although the ERI subscales were also the main contributors to the variance explained in terms of suicide risk, with ‘reward’ being negatively and ‘overcommitment’ being positively associated with suicide risk, it is nevertheless low due to the overall low variance explanation. In addition, these associations were no longer significant after the COPSOQ subscales and the CBI were included in the model.

While a very high variance explanation was achieved with the factors used for depressive symptoms, this was lower for suicidal ideation and for suicide risk. Therefore, it can be assumed that factors other than those investigated could be just as relevant, or even more relevant, as predictors of suicidal ideation and suicide risk. In a study conducted with veterinarians in Norway, both individual factors and job-related stressors were investigated as possible predictors of suicidal ideation [30]. The veterinarians surveyed most frequently reported job-related stressors as predictors of suicidal ideation. However, the regression analysis, which was also carried out, did not reveal any significant relationship between suicidal ideation and job-related stressors when both individual and job-related factors were included in the analysis. In contrast, individual factors such as being single, negative life events and mental distress were found to be predictors of suicidal ideation [30]. The fact that individual factors can cause suicidal ideation is also reflected in the Interpersonal Theory of Suicide, which states that suicidal ideation is triggered by high perceived burdensomeness and thwarted belongingness [97–99]. The extent to which perceived burdensomeness and thwarted belongingness lead to suicidal ideation is also influenced by personality traits. One study showed that people who scored high on neuroticism were more reactive to experimentally induced perceived burdensomeness and thwarted belongingness [100]. Likewise, a study in a cohort of the general population in the UK confirmed that neuroticism leads to an increased suicide risk [101]. Data collected on personality traits of veterinarians in the UK and USA show that veterinarians tend to have higher levels of neuroticism compared to the general population [102, 103]. Further research on individual factors and personality traits would lead to a more comprehensive understanding of suicidal ideation and suicide risk among veterinarians in Germany.

Some limitations need to be considered when interpreting the data that may introduce some bias into the results. Since email addresses are not mandatory data to provide to the chambers, the survey link could not be as easily and evenly distributed to all veterinarians in Germany. It may also be the case that younger colleagues were more likely to complete an online survey than older colleagues, as the proportion of younger veterinarians in the sample was higher than in the population of veterinarians in Germany [7]. Additionally, there is potential risk that individuals who are suffering from mental distress may be more likely to answer a survey than others who do not have personal experiences relating to the topic of the study. When interpreting the data, it must also be considered that the data collection was based on self-reporting. It remains to be shown whether the results can be confirmed in clinical studies. In addition, this was a cross-sectional study, so that only associations and no causal relationships can be established. Another limitation is that the data was collected in 2016. Even if current studies show that the working conditions and the mental well-being of veterinarians have not changed significantly to date [26, 27, 31], it is possible that the results of this study do not necessarily reflect the current situation of veterinarians. Furthermore, the grouping of the several fields of work into practicing and non-practicing veterinarians could lead to the results not being explicitly transferable to each field of work in detail. This could particularly affect the group of non-practicing veterinarians, since this group, in contrast to practicing veterinarians, represented a more heterogeneous group in terms of working conditions. The method used may also have limitations. While the advantage of hierarchical regression analysis is that the variance of a dependent variable can be explained by groups of associated predictor variables [104], increasing complexity of the associations between the predictor variables can make it difficult to draw definitive conclusions from the data [105].

The results of this study provide evidence that changes to working hours and compensation as well as higher appreciation in the job could improve working conditions for veterinarians and thus strengthen their mental health. However, the association of overcommitment and the demand of hiding emotions with mental health problems like depressive symptoms and suicidal ideation point to the fact that training activities and supervision should address these challenging aspects of work, both as part of prevention and intervention programs for veterinarians in the profession as well as part of the veterinary curriculum.

## Conclusions

As an increased prevalence of depression and suicidal ideation as well as an increased suicide risk were found for veterinarians in Germany [7], several demographic and job-related factors were investigated as potential risk factors in this study. Among the demographic factors, higher age and long working hours were identified as possible risk factors of suicide risk. The demographic factors were found to be negligible as risk factors of depressive symptoms and suicidal ideation. Of the job-related factors investigated in this study, low reward and high overcommitment were found to be particularly relevant for depressive symptoms, suicidal ideation, and suicide risk in German veterinarians. A positive association of depressive symptoms, suicidal ideation and suicide risk was also found with burnout, the need to hide emotions and thoughts of leaving the job. The job-related factors included in this study led to the highest overall variance explanation for depressive symptoms. Therefore, it can be concluded that these factors are more likely to be predictors of depressive symptoms than predictors of suicidal ideation or suicide risk among veterinarians in Germany.

## Supporting information

S1 Table (XLSX)
